# Potatoes as a Crop for Space Life Support: Effect of CO_2_, Irradiance, and Photoperiod on Leaf Photosynthesis and Stomatal Conductance

**DOI:** 10.3389/fpls.2019.01632

**Published:** 2019-12-19

**Authors:** Raymond M. Wheeler, Ann H. Fitzpatrick, Theodore W. Tibbitts

**Affiliations:** ^1^ NASA Exploration Research and Technology, Kennedy Space Center, Merritt Island, FL, United States; ^2^ Horticulture Department, University of Wisconsin, Madison, WI, United States

**Keywords:** potato, photosynthesis, stomatal conductance, photosynthetic photon flux, CO_2_, light, bioregenerative

## Abstract

Potatoes (*Solanum tuberosum* L.) have been suggested as a candidate crop for future space missions, based on their high yields of nutritious tubers and high harvest index. Three cultivars of potato, cvs. Norland, Russet Burbank, and Denali were grown in walk-in growth rooms at 400 and 800 µmol m^−2^ s^−1^ photosynthetic photon flux (PPF), 12-h L/12-h D and 24-h L/0 h D photoperiods, and 350 and 1,000 ppm [CO_2_]. Net photosynthetic rates (P_net_) and stomatal conductance (g_s_) of upper canopy leaves were measured at weekly intervals from 3 through 12 weeks after planting. Increased PPF resulted in increased P_net_ rates at both [CO_2_] levels and both photoperiods, but the effect was most pronounced under the 12-h photoperiod. Increased [CO_2_] increased P_net_ for both PPFs under the 12-h photoperiod, but decreased P_net_ under the 24-h photoperiod. Increased PPF increased g_s_ for both [CO_2_] levels and both photoperiods. Increased [CO_2_] decreased g_s_ for both PPFs for the 12-h photoperiod, but caused only a slight decrease under the 24-h photoperiod. Leaf P_net_ rates were highest with high PPF (800), elevated [CO_2_] (1,000), and a 12-h photoperiod, while growing the plants under continuous (24-h) light resulted in lower leaf photosynthetic rates for all combinations of PPF and [CO_2_]. The responses of leaf photosynthetic rates are generally consistent with prior published data on the plant biomass from these same studies (Wheeler *et al*., Crop Sci. 1991) and suggest that giving more light with a 24-h photoperiod can increase biomass in some cases, but the leaf P_net_ and overall photosynthetic efficiency drops.

## Introduction

Future space travel will require sustainable supplies of food, oxygen, and clean water to support human crews. For current missions like the International Space Station (ISS), food is supplied from Earth, oxygen is provided through resupply or water electrolysis, and drinking water is recovered from condensed humidity and distilled urine, along with resupply from Earth (Anderson et al., 2019). But for longer duration missions, such as living on the surface of Mars, stowage and resupply will become increasingly costly, and more regenerative life support technologies will be needed. One approach would be to grow photosynthetic organisms like plants, which could provide oxygen, remove CO_2_, and provide food (Wheeler, 2017). Plants could also be coupled to wastewater processing approaches to generate water vapor through transpiration, which then could be condensed as clean water (Wolverton et al., 1983).

A range of crops have been suggested for space life support systems, including leafy greens and vegetables as supplemental foods for early missions (Tibbitts and Alford, 1982; Massa et al., 2015; El-Nakhel et al., 2019), as well as staple type grains, legumes, and tuberous crops for more full nutrition on future missions (Tibbitts and Alford, 1982; Waters et al., 2002; Wheeler, 2017). A species found on many of these crop lists is potato, *Solanum tuberosum* L. (Tibbitts and Alford, 1982; Wheeler et al., 1986). Potatoes can be propagated vegetatively and grow well in controlled environments, even using hydroponic approaches such as nutrient film technique (Wheeler et al., 1990; Molders et al., 2012; Wheeler, 2017). In addition, when strongly induced to tuberize, potatoes can produce high yields of nutritious tubers with harvest indices as high as 0.7 to 0.8 (Tibbitts and Alford, 1982; Wheeler and Tibbitts, 1997; Wheeler, 2017; Paradiso et al., 2018).

As with many crops, total and edible biomass of potatoes can be increased with increased light and elevated [CO_2_], although the results can vary depending on the combination of environmental conditions (Demagante and Vander Zaag, 1988; Wheeler et al., 1991; Wheeler and Tibbitts, 1997; Miglietta et al., 1998; Fleisher et al., 2012; Paradiso et al., 2019). For example, proportionately greater growth from elevated [CO_2_] occurred under short photoperiods, as compared to little or no gain when plants were grown under continuous light (Wheeler et al., 1991). As with whole plant growth, net photosynthetic rates of potato canopies also increase when light and [CO_2_] are increased (Miglietta et al., 1998; Wheeler et al., 2008; Fleisher et al., 2012; Fleisher et al., 2014), yet these types of measurements require specialized chambers (Wheeler et al., 2008; Fleisher et al., 2012; Fleisher et al., 2014). More typically, photosynthetic rates of single leaves of crops such as potato have been measured using portable gas exchange systems (e.g., Chapman and Loomis, 1953; Dwelle et al., 1981; Dwelle et al., 1982; Dwelle, 1985; Sicher and Bunce, 2001; Lawson et al., 2002; Paradiso et al., 2018). As with whole canopy measurements, these single leaf measurements of photosynthesis typically showed increased rates when light and CO_2_ were increased (Dwelle et al., 1981; Dwelle et al., 1982; Sicher and Bunce, 2001; Bunce, 2003; Kaminski et al., 2014; Paradiso et al., 2018). But as with some other species, potato leaves can acclimate to prolonged elevated [CO_2_] and leaf Rubisco protein and total nitrogen levels can drop (Sicher and Bunce, 2001; Leakey et al., 2012).

Interacting effects of [CO_2_] and light on photosynthesis to light can be complex (Nilwik et al., 1982; Lakso et al., 1984; Hand et al., 1993) and there are few studies examining these interactions for potato. Chapman and Loomis (1953) reported increased photosynthetic rates for potato leaves under elevated [CO_2_] in field settings, and showed that light saturation could be increased by [CO_2_] enrichment from 3,000 foot-candles [approximately 585 µmol m^−2^ s^−1^ photosynthetic photon flux (PPF)], to 4,200 foot-candles (approximately 820 µmol m^−2^ s^−1^ PPF) at 2X ambient [CO_2_], and to 5,200 foot-candles (approximately 1,015 µmol m^−2^ s^−1^ PPF) at 5X ambient [CO_2_] (irradiance conversions based on Deitzer, 1994). Extrapolating the inflections of their light-[CO_2_] response curves forms a straight line implying that significantly higher photosynthetic rates might be attainable. Ku et al. (1977) reported that an irradiance of 850 µmol m^−2^ s^−1^ (~ ½ full sunlight) was saturating for potato leaves at ambient [CO_2_], but doubling the [CO_2_] could approximately double the photosynthetic rate. Others have reported maximum rates in field grown potatoes to occur near 1,200 µmol m^−2^ s^−1^ PPF, and that photosynthetic rates were closely correlated to stomatal conductance at high irradiance levels in the field (Dwelle, 1985). Maximum gross photosynthetic rates for some potato cultivars under full sunlight (~2,000 µmol m^−2^ s^−1^ PPF) ranged from 50 to 60 mg CO_2_ dm^−2^ h^−1^, or about 32–38 µmol CO_2_ m^−2^ s^−1^ (Dwelle, 1985).

In an earlier paper, we reported on the effects of [CO_2_], PPF, and photoperiod with potato, where total biomass increased in response to increased [CO_2_] and PPF with a 12-h photoperiod, and but [CO_2_] had little or no beneficial effect under a 24-h photoperiod (Wheeler et al., 1991). Likewise, tuber yields were increased with increasing [CO_2_] and PPF under a 12-h photoperiod, but neither [CO_2_] nor PPF affected tuber yield under 24-h continuous lighting. This range of effects of [CO_2_], PPF, and photoperiod on potato tuber and total biomass yields suggest that primary physiological functions, like photosynthetic rates might be affected in complex or interacting ways by these same environmental factors. Here we report on leaf photosynthetic rates and stomatal conductance from these same studies. We hypothesized that the trends in total biomass can be predicted by measurements of leaf net photosynthetic rates. Note that these studies were conducted in 1987 at the University of Wisconsin Biotron (Madison, WI, US), and the ambient [CO_2_] levels were approximately 350 ppm as compared to ~410 ppm in 2019, 32 years later.

## Materials and Methods

**Figure 1:**
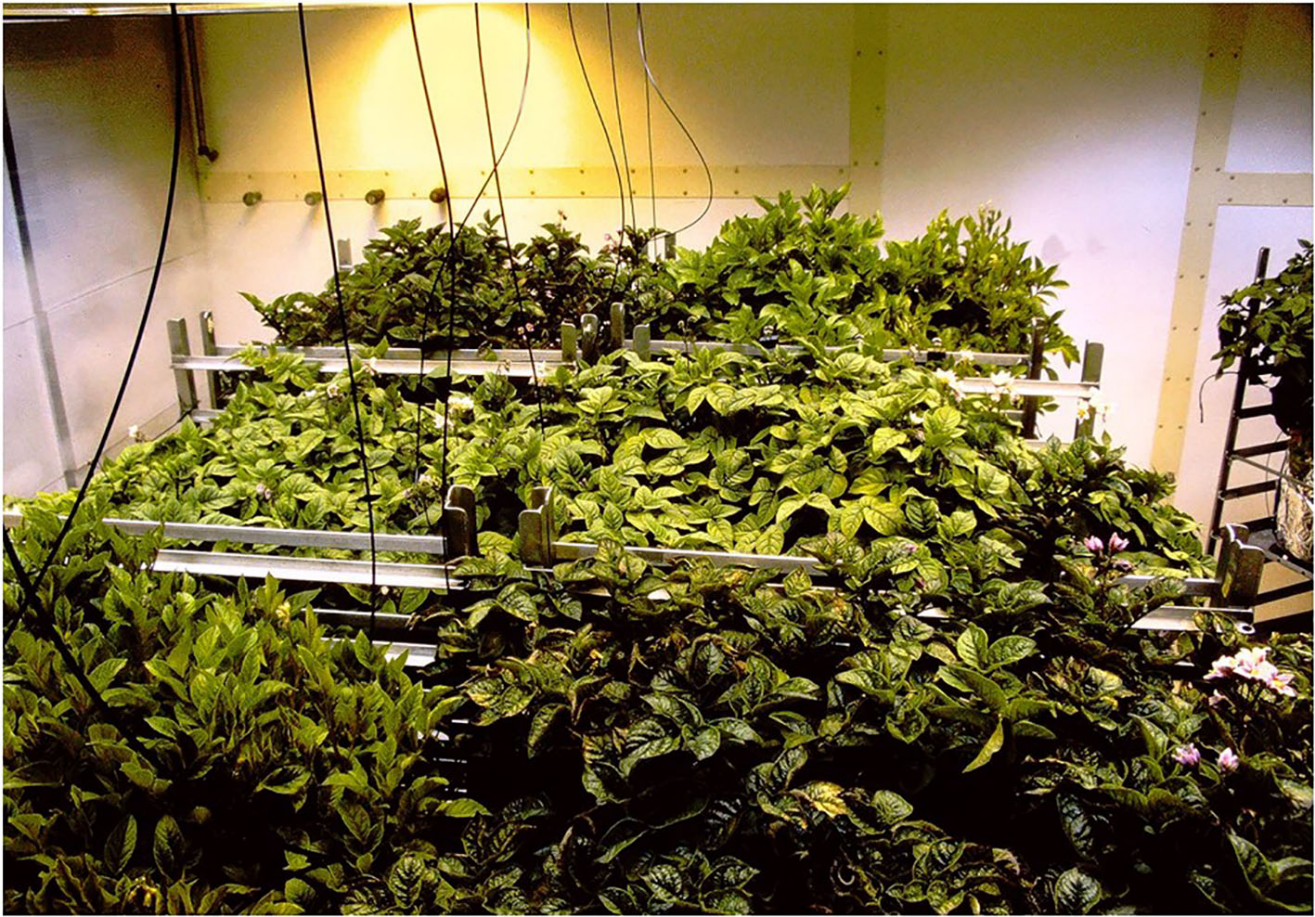
Large walk-in grown chamber at the University of Wisconsin Biotron used for study of potatoes grown at different levels of CO_2_, photosynthetic photon flux (PPF), and photoperiods. Three cultivars were included: Russet Burbank, Norland, and Denali. Each cultivar had three plants placed on two mobile carts positioned under 400 µmol m^−2^ s^−1^ PPF and two under 800 µmol m^−2^ s^−1^ PPF (total of six plants per treatment). Cart positions were changed at weekly intervals to reduce position effects.

Propagation and cultural techniques for the potato plants (*S. tuberosum* L. cvs. Norland, Russet Burbank, and Denali) have been described in detail previously (Wheeler and Tibbitts, 1987). In brief, plants were started from *in vitro* propagated stem cuttings planted in 30-cm (19-L) pots containing peat-vermiculite (50:50 vol.) potting mix. Approximately 2/3 of the plantlet stem was buried in the potting medium and the above ground portion was covered with a glass beaker for 3 days to allow the plantlet to acclimate. All pots were watered to excess four times daily with a complete nutrient solution (Wheeler and Tibbitts, 1987). Studies were carried out in walk-in growth rooms at the University of Wisconsin Biotron (**Figure 1**). Environmental variables examined included: 1) carbon dioxide—ambient (~350 ppm—nominal ambient in 1987) and elevated (1,000 ppm); 2) photoperiod—12-light/12-h dark and 24-h light/0-h dark (i.e., continuous light), and 3) irradiance—400 and 800 µmol m^−2^ s^−1^ PPF from a mixture of high pressure sodium and metal halide lamps (Wheeler et al., 1991). The experiment was set up as factorial design with eight different combinations of [CO_2_], photoperiod, and irradiance. Two growth rooms were used simultaneously for the study, where the [CO_2_] was controlled to 350 ppm in one room and 1,000 ppm in the second room by supplementing the chamber air with pure CO_2_. Within each [CO_2_] controlled chamber, plants were placed on carts positioned under lamps that provided a zone of 400 µmol m^−2^ s^−1^ PPF or a zone of 800 µmol m^−2^ s^−1^ PPF. This was achieved by placing twice as many lamps over the 800 µmol m^−2^ s^−1^ zone. Heights of the carts were adjustable to maintain the target PPF values at the top of the plant canopy. Three pots were placed on each cart, and there were two carts (total of six plants) for each cultivar in each PPF zone (**Figure 1**) (Wheeler et al., 1991). Carts were moved to a new position each week to minimize position effects in the growth rooms. The growth rooms were both set to a 12-h photoperiod and plants were grown for 90 days and then harvested (Wheeler et al., 1991). Following this, each growth room was replanted, the photoperiod was set to a 24-h (continuous light) and plants were again grown for 90 days. Air temperature and relative humidity were maintained constant at 16°C and 70% for all tests.

### Net Photosynthesis

Leaf photosynthetic (CO_2_ assimilation) rates (P_net_) were measured with a Li-Cor LI-6000 portable photosynthesis system at 3, 4, 5, 6, 8, 10, and 12 weeks after planting. A total of 12 measurements (two per plant from three plants on two separate carts) were taken on exposed, fully-expanded terminal or penultimate leaflets for each cultivar at each date under each environmental combination. Leaves were measured *in situ* with the chamber lighting. Leaves were positioned to be as close as possible to the target PPFs of 400 and 800 µmol m^−2^ s^−1^, but incident PPF was reduced approximately 10–15% by the Plexiglas of the cuvette. Approximately 13 cm^2^ of leaf tissue were enclosed in a 330-ml clear Plexiglas cuvette for the measurements. After enclosing the leaf, the [CO_2_] draw-down of the cuvette was logged in a series of ten 2- or 4-s intervals depending on the rate of photosynthesis. This draw-down rarely exceeded 30 ppm below the starting [CO_2_] concentration and each measurement could be completed in less than 1 min. A flow rate of approximately 20 ml s^−1^ was maintained between the infrared gas analyzer and the cuvette to provide uniform mixing and purging between measurements.

**Figure 2:**
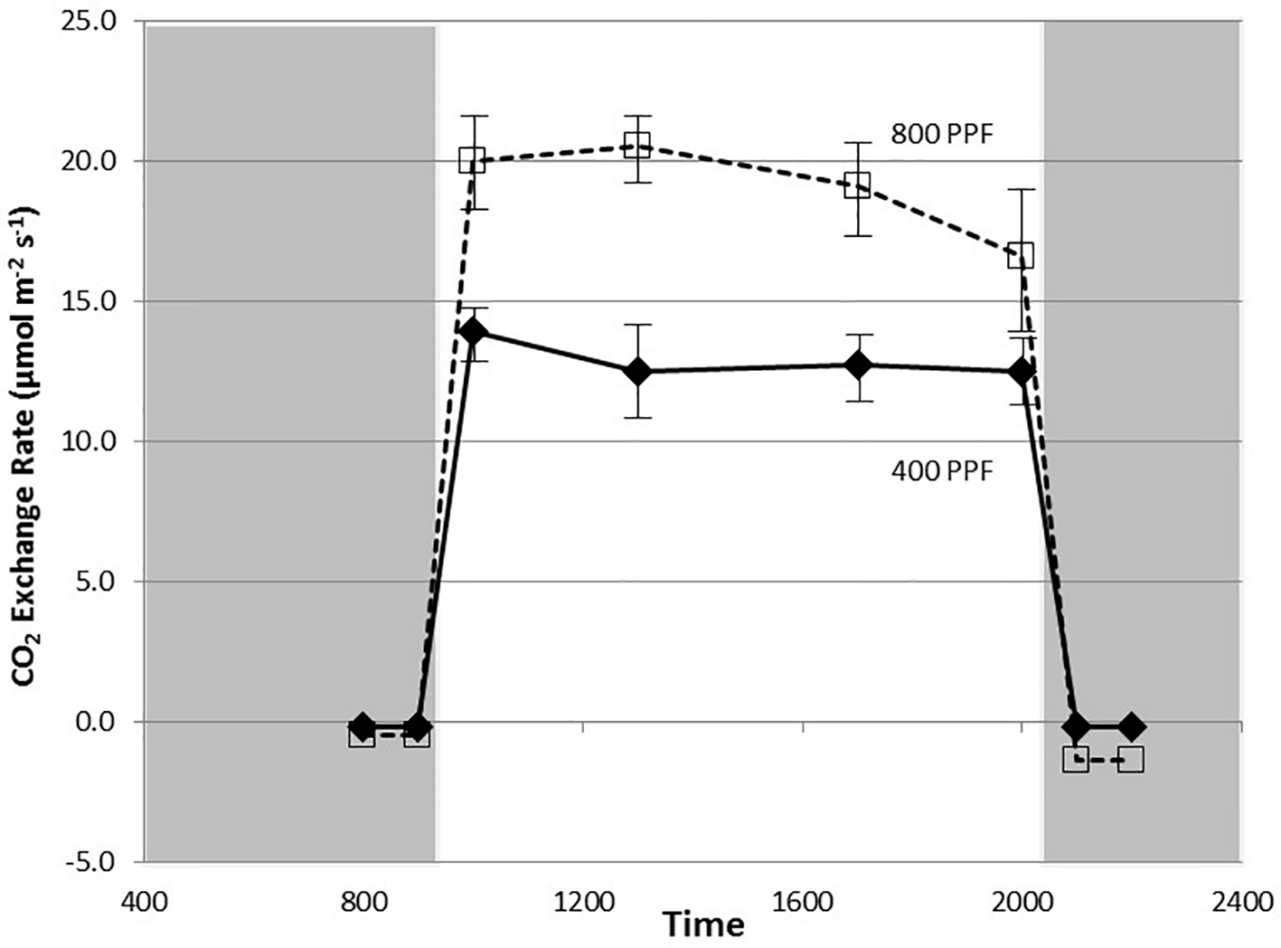
Diurnal plot of net CO_2_ exchange rates for young, fully expanded potato leaves of cv. Denali grown under a 12-h light and 12-h dark photoperiod at 350 ppm [CO_2_]. Plants were grown at either 400 or 800 µmol m^−2^ s^−1^ photosynthetic photon flux (PPF). Each point represents n = 12 measurements from separate leaves, with standard deviation bars indicated. Positive values of CO_2_ exchange in the light represent net photosynthesis and negative values in the dark represent respiration.

Typically a set of measurements lasted 2 h per room. For the treatments involving 12-h light and 12-h dark photoperiods, measurements began 5 h into the light thereby straddling the middle of the light period (**Figure 2**). For 1 day prior to any photosynthetic or stomatal conductance measurements, entrance into the growth rooms was restricted to avoid and transient [CO_2_] fluctuations. During all measurements, gas masks connected by hoses to a vacuum pump outside the room were worn to remove exhaled breath from the rooms (Wheeler and Tibbitts, 1989). CO_2_ concentrations could thus be held constant during the ~2 h required for the measurements.

### Stomatal Conductance

**Figure 3:**
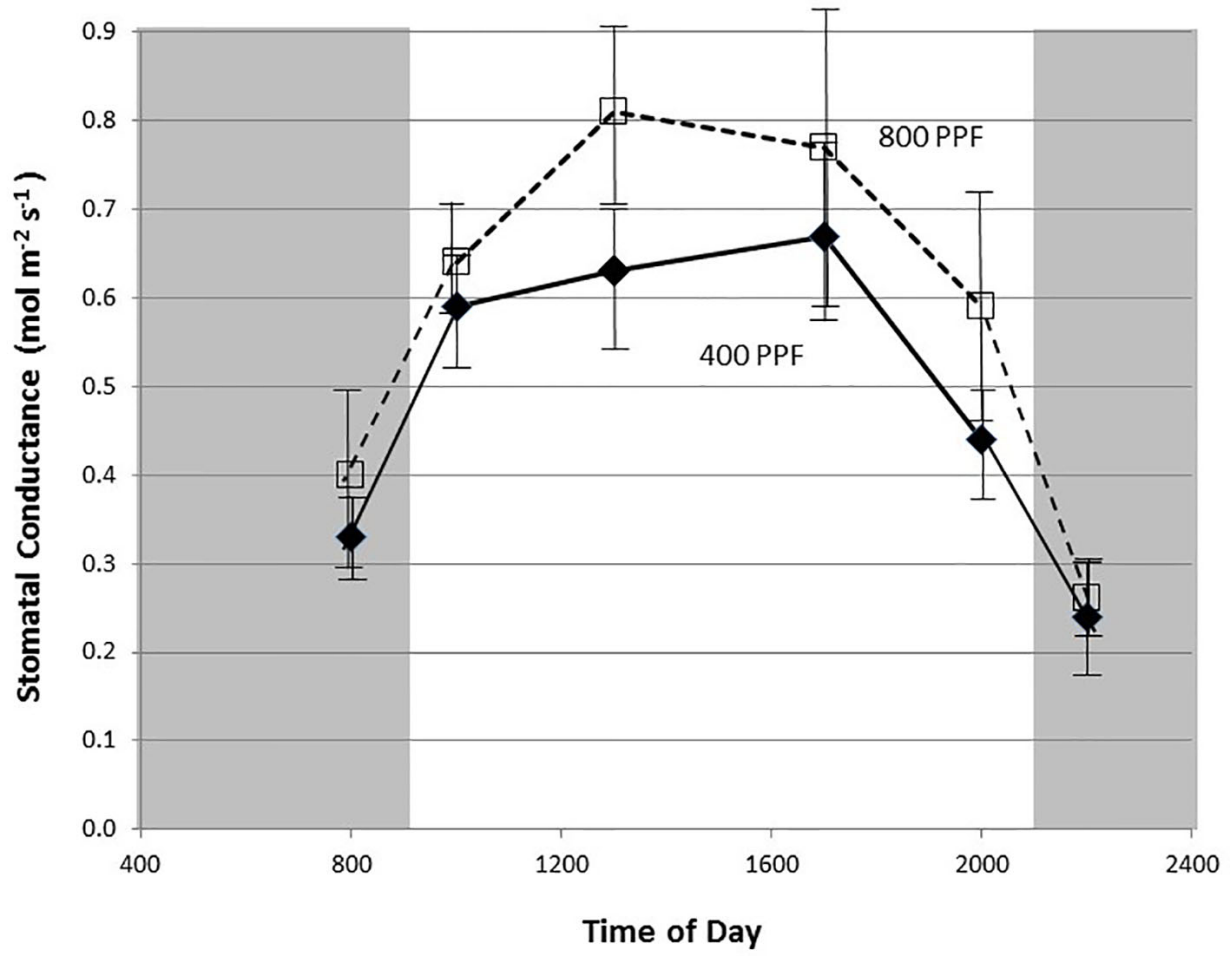
Diurnal plot of leaf stomatal conductance rates for young, fully expanded potato leaves of cv. Denali grown under a 12-h light and 12-h dark photoperiod at 350 ppm [CO_2_]. Plants were grown at either 400 or 800 µmol m^−2^ s^−1^ photosynthetic photon flux (PPF). Each point represents n = 12 measurements from separate leaves with standard deviation bars indicated.

Stomatal conductance (g_s_) data were logged simultaneously with CO_2_ assimilation data by the LI-6000 portable photosynthesis system. A total of 12 measurements (two per plant from three plants on two separate carts) were taken on exposed, fully-expanded terminal or penultimate leaflets for each cultivar at each date under each environmental combination. Stomatal conductance measurements for plants grown under 12-photoperiods were taken approximately in the middle of light cycle. Measurements taken across the full photoperiod showed a clear diurnal rhythm with rates typically peaking in the middle of the photoperiod (**Figure 3**), thus stomatal conductance data measured in these tests likely represented peak rates for the plants grown under a 12-h photoperiod. Previous studies showed no measurable differences in conductance or CO_2_ exchange rates based on time of day for plants grown under continuous light (Wheeler and Tibbitts, 1989).

Because of the decision to maintain a rapid air flow to the cuvette for rapid purging of the system, cuvette humidity decreased during the logging sequence. Air flow for the LI-6000 device used in this study was cycled through a desiccant column before entry to the IRGA and subsequently returned to the cuvette. Equations used by the LI-6000 accounted for humidity changes in conductance calculations, however in practice, we found that such decreases in humidity resulted in slightly higher estimates of conductance in comparison to measurement at steady state humidity. This was likely a result of water desorption from the cuvette walls and tubing during humidity drops, which the machine interpreted as transpired water. Assuming that such errors were relatively constant, the uniformity in our procedures should allow relative comparisons of conductance between treatments. More updated versions of the Li-Cor portable photosynthesis systems (e.g., the 6200, 6400, 6800 models) maintain near steady state humidity and are not affected by this phenomenon (McDermitt, 1990).

### Statistical Analysis

Data averaged over multiple dates during growth and development were compared by calculating standard errors of the means. Data gathered at two developmental stages, i.e., 28 days and 70 days after planting, were compared using analysis of variance using a 2 x 2 x 2 x 3 analysis design (two [CO_2_] concentrations, two PPF levels, two photoperiods, and three cultivars). Main effects, two-way interactions, and three-way interactions were compared at 95% confidence (P value = 0.05) and 99% confidence (P value = 0.01).

## Results

Measurement of leaf CO_2_ exchange rates for cv. Denali leaves before, during, and after a 12-h photoperiod showed that leaf P_net_ rates rose rapidly when the lights came on (09:00) and maintained relatively even rate across the light period (**Figure 2**). Each point shown in **Figure 2** is the average of measurements from 12 different leaves. Prior to the light cycle and after the initiation of the dark period (21:00), CO_2_ exchange rates were slightly negative indicating a net CO_2_ efflux from leaf respiration (**Figure 2**). The dark period respiration rates were slightly greater for the 800 PPF plants than the 400 plants, possibly related to greater accumulation of photo-assimilates in the leaves grown under higher light. These measurements were taken at 28 days after planting at the 350 ppm [CO_2_] level. Raising the PPF from 400 to 800 µmol m^−2^ s^−1^ increased leaf P_net_ by 47% across the light period. Simultaneous measurements of stomatal conductance (g_s_) showed an increase at the beginning of the photoperiod and with peak rates occurring in the middle of the photoperiod (**Figure 3**). Each point shown in **Figure 3** is the average of measurements from 12 different leaves. Conductance rates dropped toward the end of the photoperiod and continued to decrease with the onset of the dark cycle, indicating a typical circadian rhythm effect on stomata (Holmes and Klein, 1986). Raising the PPF from 400 to 800 µmol m^−2^ s^−1^ increased g_s_ by 21% across the across the photoperiod, although standard deviations for all but 13:00 measurements overlapped (**Figure 3**). The greatest relative difference occurred in the middle of the light cycle (**Figure 3**).

**Figure 4:**
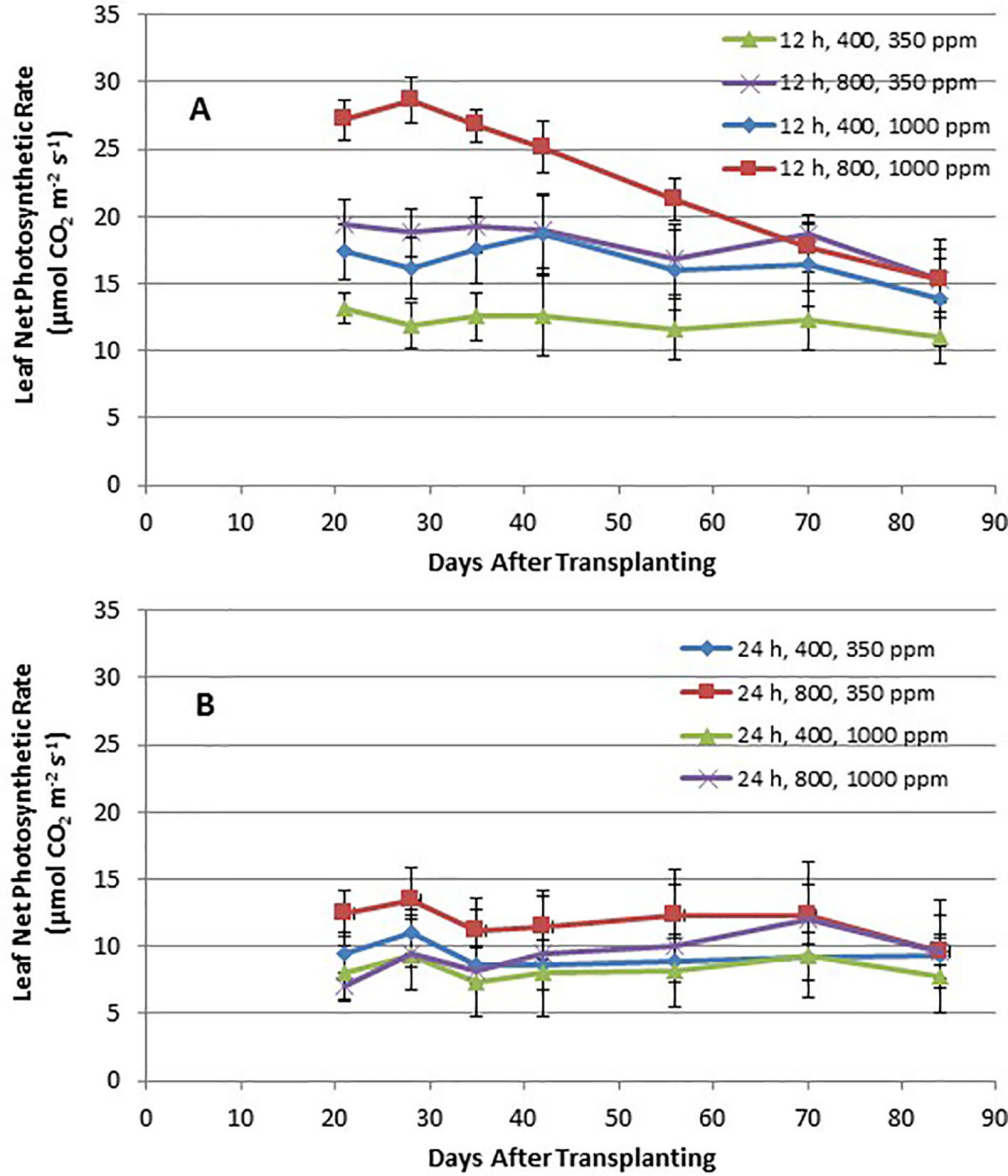
**(A** and **B)**. Time course plot of leaf net photosynthetic rates for upper canopy potato leaves grown under different combinations of photoperiod, [CO_2_], and PPF. Data show combined values of 12 measurements each for cvs. Denali, Russet Burbank, and Norland (total of 36 measurements) for each point. Panel **(A)** shows data from plants grown under a 12-h light/12-h dark photoperiod, with measurement taken around the middle of the photoperiod. Panel **(B)** shows data from plants grown under 24-h or continuous light. Standard deviations for each set of 36 measurements (each point) are indicated.

**Figure 5:**
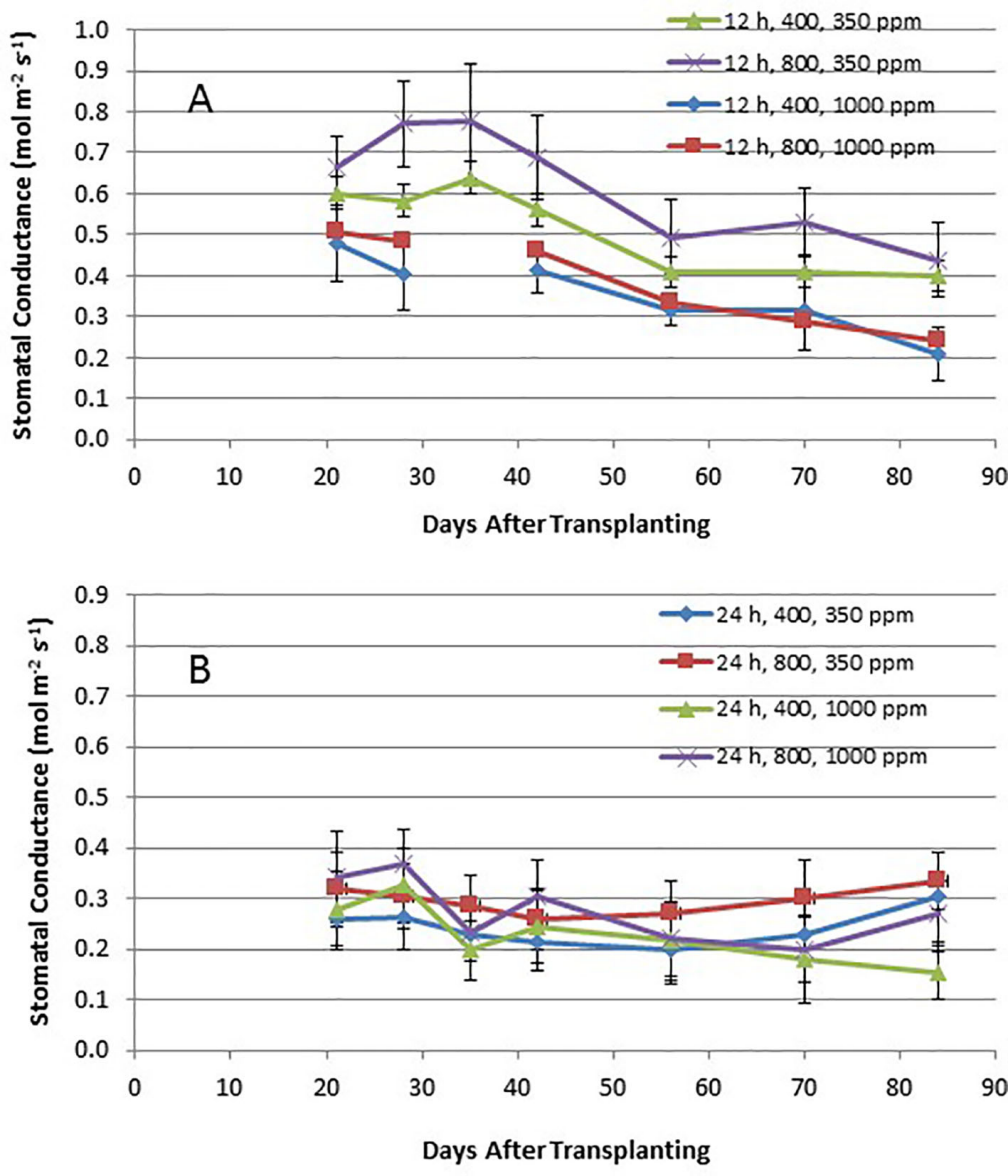
**(A** and **B)**. Time course plot of stomatal conductance for upper canopy potato leaves grown under different combinations of photoperiod, [CO_2_], and PPF. Data show combined values of 12 measurements each for cvs. Denali, Russet Burbank, and Norland (total of 36 measurements) for each point. Panel **(A)** shows data from plants grown under a 12-h light/12-h dark photoperiod, with measurements taken around the middle of the photoperiod. Panel **(B)** shows data from plants grown under 24-h (continuous) light. Standard deviations for each set of 36 measurements (each point) are indicated.

Time course plots of the leaf net photosynthesis (P_net_) rates from day 21 through day 84 for the 12-h photoperiod and 24-h photoperiod grown plants under the various combinations of PPF and [CO_2_] are shown in **Figures 4A**, **B**. Upper canopy leaves of plants grown under 12-h photoperiods showed higher photosynthetic rates than leaves under 24-h (continuous) light throughout all of the growth cycle (**Figures 4A**,** B**). P_net_ rates were highest for leaves under 12-h photoperiods with elevated [CO_2_] (1,000 ppm) and high PPF (800 µmol m^−2^ s^−1^), but these rates dropped with age (**Figure 4A**). Time course plots of stomatal conductance (g_s_) rates from day 21 through day 84 for the 12-h photoperiod and 24-h photoperiod grown plants are shown in **Figures 5A**, **B**. Mid-day g_s_ rates for 12-h photoperiod plants were higher than rates of 24-h photoperiod for most of the growth cycle, but the 12-h plants showed a clear diurnal rhythm in their stomatal control (**Figure 3**), while g_s_ rates for 24-h (continuous light) plants showed little change across the day (data not shown). Mid-day g_s_ rates for 12-h plants grown at 350 ppm [CO_2_] were higher throughout most of growth cycle compared to 12-h plants grown under 1,000 ppm [CO_2_].

Using analysis of variance for measurements taken from plants at 28 days after planting, leaf P_net_ rates taken at the middle of the 12-h photoperiod showed significant main effects for [CO_2_] (i.e., 350 *vs.* 1,000 ppm), PPF (i.e., 400 *vs.* 800 µmol m^−2^ s^−1^), and photoperiod (i.e., 12-h *vs.* 24-h photoperiods), but no significant differences among cultivars (**Table 1**). Significant two-way interactions were also apparent for [CO_2_] x photoperiod, and PPF x photoperiod, and a significant three-way interaction occurred for [CO_2_] x PPF x photoperiod. At 70 days after planting, significant main effects were apparent for PPF and photoperiod for leaf photosynthetic rates, but the [CO_2_] effect was not significant, and there were no significant interacting effects (**Table 2**).

**Table 1:** Net photosynthetic rates of potato leaves under different photoperiods, CO_2_, and photosynthetic photon flux levels (28 days after planting).

Cultivar	CO_2_ (ppm)	Photoperiod (h)			
		12		24	
		PPF (µmol m^−2^ s^−1^)			
		400	800	400	800
		(µmol CO_2_ m^−2^ s^−1^)			
Norland	350	12.59	19.34	12.98	13.23
	1,000	16.32	29.03	10.84	12.48
Russet Burbank	350	10.50	16.64	10.09	13.16
	1,000	14.43	26.23	9.32	7.91
Denali	350	12.50	20.37	10.23	13.96
	1,000	17.66	30.57	7.96	7.91
CO_2_			*		
PPF			**		
Photoperiod			**		
Cultivar (cv.)			NS		
CO_2_ x PPF			NS		
CO_2_ x Photoper.			**		
CO_2_ x cv.			NS		
PPF x Photoper.			**		
PPF x cv.			NS		
Photoperiod x cv.			NS		
CO_2_ x PPF x Photoper.			*		
CO_2_ x PPF x cv.			NS		
CO_2_ x Photoper. x cv.			NS		
PPF x Photoper. x cv.			NS		

*,** significant at P = 0.05 and P = 0.01, respectively. NS, not significant.

**Table 2:** Net photosynthetic rates of potatoes under different photoperiods, CO_2_, and photosynthetic photon flux levels (70 days after planting).

Cultivar	CO_2_ (ppm)	Photoperiod (h)			
		12		24	
		PPF (µmol m^−2^ s^−1^)			
		400	800	400	800
		(µmol CO_2_ m^−2^ s^−1^)			
Norland	350	12.23	19.48	7.50	11.18
	1,000	16.37	16.96	7.71	15.37
Russet Burbank	350	11.75	16.32	9.39	10.23
	1,000	15.23	16.18	10.36	10.09
Denali	350	12.73	20.25	10.84	15.64
	1,000	17.73	19.80	9.82	10.57
CO_2_			NS		
PPF			*		
Photoperiod			*		
Cultivar (cv.)			NS		
CO_2_ x PPF			NS		
CO_2_ x Photoper.			NS		
CO_2_ x cv.			NS		
PPF x Photoper.			NS		
PPF x cv.			NS		
Photoperiod x cv.			NS		
CO_2_ x PPF x Photoper.			NS		
CO_2_ x PPF x cv.			NS		
CO_2_ x Photoper. x cv.			NS		
PPF x Photoper. x cv.			NS		

*, significant at P = 0.05 and P = 0.01, respectively. NS, not significant.

Analysis of variance of g_s_ measurements at 28 days after planting showed a significant main effect for [CO_2_], PPF, and photoperiod but not cultivar, and a significant two-way interaction for [CO_2_] x photoperiod (**Table 3**). At 70 days after planting, g_s_ measurements showed significant main effects [CO_2_], PPF, photoperiod, and cultivar, and significant two-way interactions for [CO_2_] x PPF, [CO_2_] x photoperiod, and photoperiod by cultivar (**Table 4**).

**Table 3:** Stomatal conductance rates of potato leaves under different photoperiods, CO_2_, and photosynthetic photon flux levels (28 days after planting).

Cultivar	CO_2_ (ppm)	Photoperiod (h)			
		12		24	
		PPF (µmol m^−2^ s^−1^)			
		400	800	400	800
		(mol m^−2^ s^−1^)			
Norland	350	0.604	0.824	0.297	0.311
	1,000	0.418	0.514	0.331	0.214
Russet Burbank	350	0.513	0.678	0.239	0.258
	1,000	0.342	0.454	0.243	0.367
Denali	350	0.632	0.809	0.251	0.251
	1,000	0.453	0.484	0.401	0.525
CO_2_			*		
PPF			*		
Photoperiod			**		
Cultivar (cv.)			NS		
CO_2_ x PPF			NS		
CO_2_ x Photoper.			**		
CO_2_ x cv.			NS		
PPF x Photoper,			NS		
PPF x cv.			NS		
Photoperiod x cv.			NS		
CO_2_ x PPF x Photoper.			NS		
CO_2_ x PPF x cv.			NS		
CO_2_ x Photoper. x cv.			NS		
CO_2_ PPF x Photoper. x cv.			NS		

*,** significant at P = 0.05 and P = 0.01, respectively. NS, not significant.

**Table 4:** Stomatal conductance rates of potato leaves under different photoperiods, CO_2_, and photosynthetic photon flux levels (70 days after planting).

Cultivar	CO_2_ (ppm)	Photoperiod (h)			
		12		24	
		PPF (µmol m^−2^ s^−1^)			
		400	800	400	800
		(mol m^−2^ s^−1^)			
Norland	350	0.457	0.617	0.617	0.281
	1,000	0.340	0.246	0.152	0.155
Russet Burbank	350	0.361	0.417	0.189	0.205
	1,000	0.230	0.252	0.132	0.173
Denali	350	0.412	0.559	0.559	0.420
	1,000	0.371	0.367	0.258	0.265
CO_2_			_**_		
PPF			*		
Photoperiod			*		
Cultivar (cv.)			**		
CO_2_ x PPF			*		
CO_2_ x Photoper.			*		
CO_2_ x cv.			NS		
PPF x Photoper,			NS		
CO_2_ x cv.			NS		
Photoperiod x cv.			*		
CO_2_ x PPF x Photoper.			NS		
CO_2_ x PPF x cv.			NS		
PPF x Photoper. x cv.			NS		
PPF x Photoper. x cv.			NS		

*,** significant at P = 0.05 and P = 0.01, respectively. NS, not significant.

**Figure 6:**
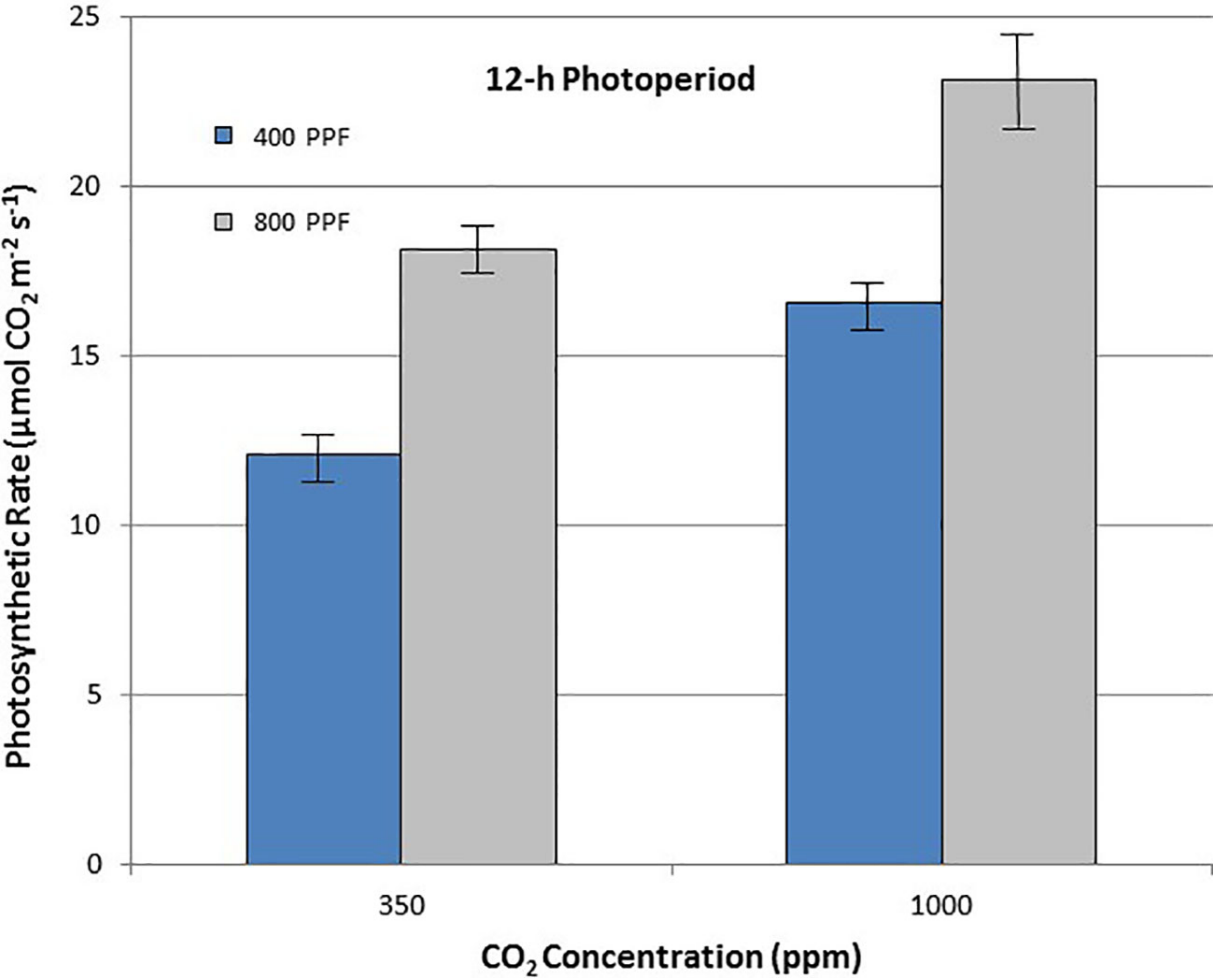
Comparison of time-averaged leaf net photosynthetic rates taken at 21, 28, 35, 42, 56, 70, and 84 days after planting for all cultivars grown under a 12-h photoperiod at two [CO_2_] concentrations, and two photosynthetic photon flux (PPF) levels. A total of n = 36 measurements were taken at each of the 7 dates, for a total 252 total measurements. Error bars indicate the standard error of the mean for average measurements at each date.

**Figure 7:**
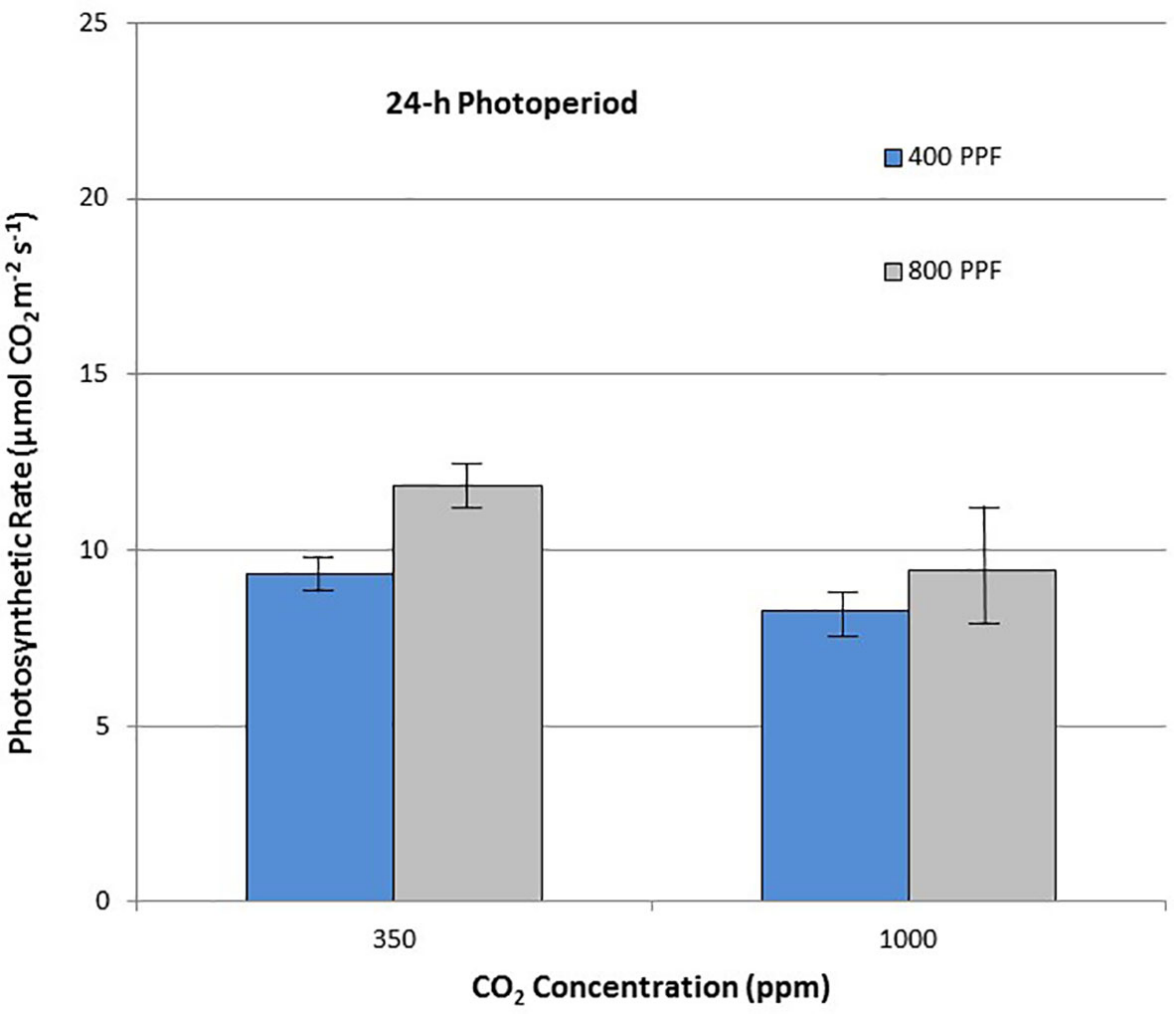
Comparison of time-averaged leaf net photosynthetic rates taken at 21, 28, 35, 42, 56, 70, and 84 days after planting for all cultivars grown under 24-h (continuous) light at two [CO_2_] concentrations, and two photosynthetic photon flux (PPF) levels. A total of n = 36 measurements were taken at each of the 7 dates, for a total 252 total measurements. Error bars indicate the standard error of the mean for average measurements at each date.

When comparing leaf net photosynthesis for all ages and all cultivars, increasing the [CO_2_] from 350 to 1,000 ppm increased P_net_ rates the most under the 12-h photoperiod, with an overall increase of 36% occurring at 400 µmol m^−2^ s^−1^ PPF and 27% at 800 µmol m^−2^ s^−1^ PPF (**Figure 6**). In contrast, increasing [CO_2_] from 350 to 1,000 ppm under the 24-h (continuous) light treatment decreased leaf net photosynthetic rates by 11% at 400 µmol m^−2^ s^−1^ PPF and 20% at 800 µmol m^−2^ s^−1^ PPF, although rates had overlapping standard errors (**Figure 7**).

When comparing photosynthetic rates for all ages and all cultivars, but in this case for PPF effects, the greatest increase occurred with 12-h plants grown at 350 ppm [CO_2_], showing a 49% increase in P_net_, and plants grown at 12-h with 1,000 ppm [CO_2_] showing a 40% increase (**Figure 6**). Plants grown under the 24-h (continuous) light also showed increased P_net_ rates in response to increased PPF, but only 27% at 350 ppm [CO_2_] and 12% increase at 1,000 ppm [CO_2_], which was not significantly different based on standard errors (**Figure 7**).

**Figure 8:**
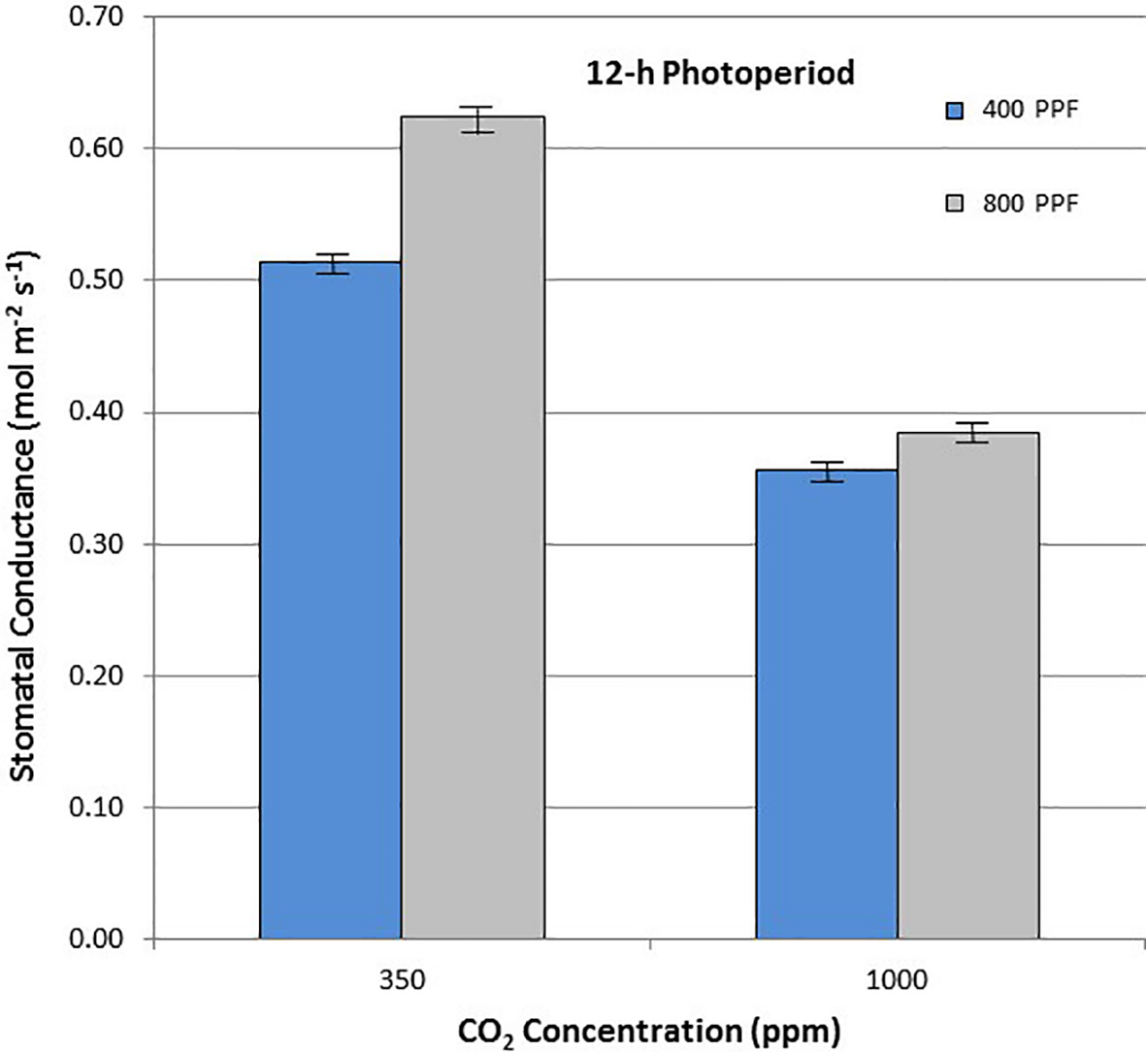
Comparison of time-averaged stomatal conductance rates taken at 21, 28, 35, 42, 56, 70, and 84 days after planting for all cultivars grown under a 12-h photoperiod at two [CO_2_] concentrations, and two photosynthetic photon flux (PPF) levels. A total of n = 36 measurements were taken at each of the 7 dates, for a total 252 total measurements. Error bars indicate the standard error of the mean for average measurements at each date.

**Figure 9:**
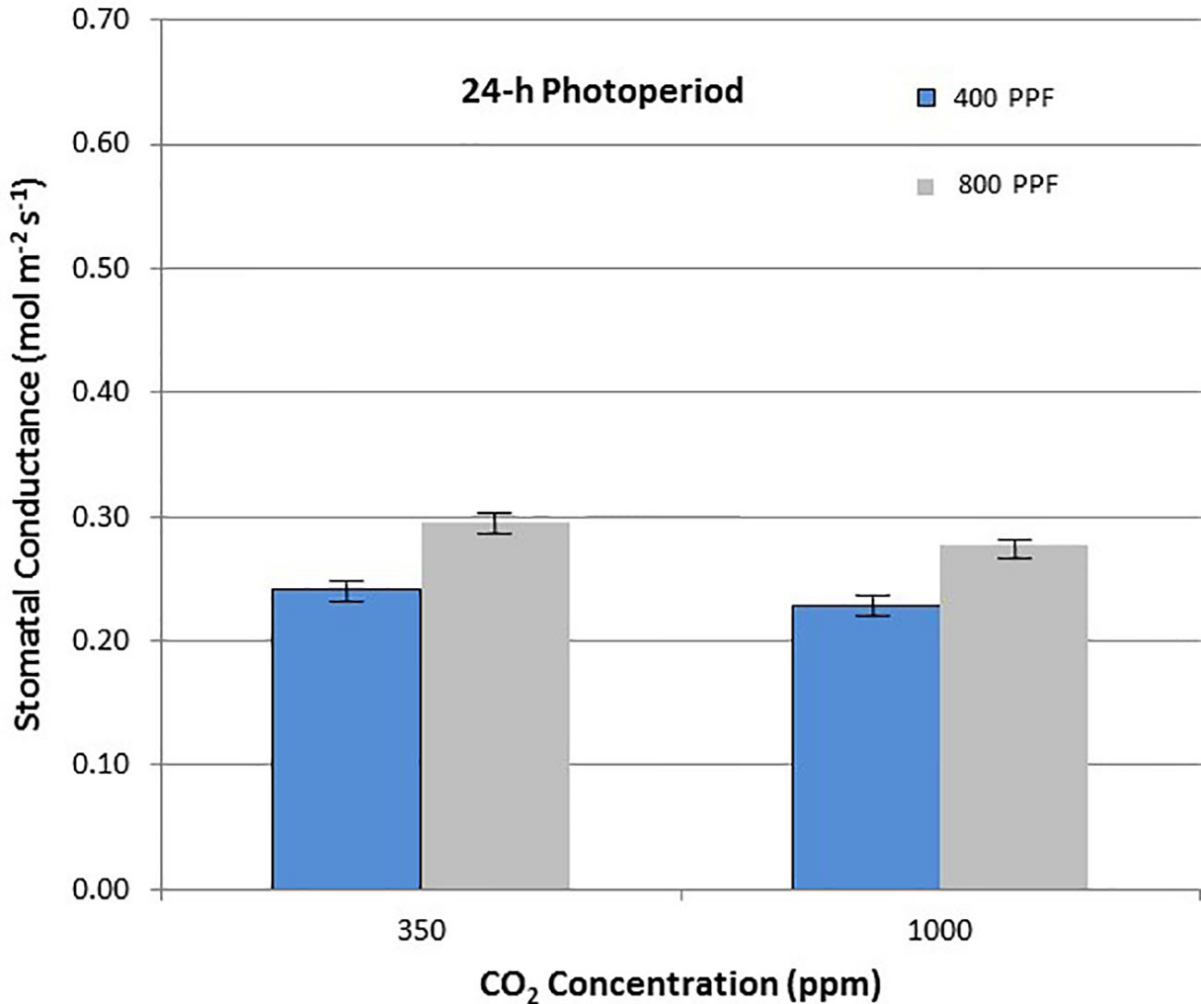
Comparison of time-averaged stomatal conductance rates taken at 21, 28, 35, 42, 56, 70, and 84 days after planting for all cultivars grown under 24-h (continuous) light at two [CO_2_] concentrations, and two photosynthetic photon flux (PPF) levels. A total of n = 36 measurements were taken at each of the 7 dates, for a total 252 total measurements. Error bars indicate the standard error of the mean for average measurements at each date.

When comparing stomatal conductance (g_s_) for all ages and all cultivars, increasing the [CO_2_] from 350 to 1,000 ppm under the 12-h photoperiod decreased g_s_ by 29% at 400 µmol m^−2^ s^−1^ PPF by 37% at 800 µmol m^−2^ s^−1^ PPF (**Figure 8**). Under 24-h continuous light, increasing the [CO_2_] from 350 to 1,000 ppm decreased g_s_ by 8% at the 400 µmol m^−2^ s^−1^ PPF, and by 7% at 800 µmol m^−2^ s^−1^ PPF (**Figure 9**).

When comparing g_s_ for all ages and all cultivars but in this case for PPF effects, increasing the PPF from 400 to 800 µmol m^−2^ s^−1^ increased g_s_ for 12-h plants by 22% at 350 ppm [CO_2_] and by 8% at 1,000 ppm [CO_2_] (**Figure 8**). Under 24-h continuous light, increasing the PPF from 400 to 800 µmol m^−2^ s^−1^ increased g_s_ by 25% at 350 ppm [CO_2_] and by 23% at 1,000 ppm [CO_2_] (**Figure 9**).

## Discussion

Time course measurements of leaf photosynthesis and conductance showed the highest P_net_ rates for 12-h photoperiod, 1,000 ppm [CO_2_], and 800 µmol m^−2^ s^−1^ PPF early in growth, after which P_net_ rates of the upper canopy leaves declined with time, which is consistent with reports in the literature (Vos and Oyarzun, 1987; Olesinski et al., 1989; Tekalign and Hammes, 2005; Timlin et al., 2006; Fleisher et al., 2014). P_net_ rates for other combinations of photoperiod, [CO_2_] and PPF tended to remain relatively constant between 21 and 84 days after planting (**Figure 4**). Time course measurements for stomatal conductance, g_s_, showed the highest rates early in growth under all of the 12-h photoperiod treatments, followed by a graduate decline with age (Vos and Oyarzun, 1987). In contrast, g_s_ for upper canopy leaves for all the 24-h treatments did not change much with age (**Figure 5**). No measurements of P_net_ or g_s_ were taken at 90 days, when plants were harvested and upper canopy leaves had begun to senesce, but it is likely that P_net_ or g_s_ rates for most treatments would have decreased with the onset of upper canopy leaf senescence.

When plants were grown under a 12-light/12-h dark photoperiod, stomatal conductance showed a clear diurnal rhythm of opening in the morning, peaking mid-day, and then beginning to close with the onset of dusk (**Figure 3**). These rhythms were likely controlled by a circadian cycle entrained to the photoperiod (Holmes and Klein, 1986). This pattern of stomatal opening and closing occurred at both PPFs, and related studies showed that elevating the [CO_2_] from 400 to 1,000 ppm reduced stomatal conductance in potato leaves, and that diurnal rhythms persist (Wheeler et al., 1999). Clear diurnal patterns for conductance have been reported from in-field grown potatoes (cv. Russet Burbank) and conductance levels rose linearly when PPF was increased from 400 to 2,000 µmol m^−2^ s^−1^ (Dwelle et al., 1982).

A statistical comparison of data gathered at 28 after planting showed that [CO_2_], PPF, and photoperiod all had significant effects on P_net_, and that the effects of both [CO_2_] and PPF interacted with photoperiod (**Table 1**). At 70 days after planting, PPF and photoperiod still had significant effects on P_net_, but [CO_2_] was not significant, and there were no significant interactions among factors (**Table 2**). This indicates that plant age and stage of development should be considered when comparing the influence of environmental factors such as light and [CO_2_] leaf photosynthetic rates of potato.

As with P_net_, stomatal conductance at 28 days after planting was affected by [CO_2_], PPF, and photoperiod, and the influence of [CO_2_] depended on the photoperiod (**Table 3**). Unlike P_net_, g_s_ at 70 days after planting was also dependent on the cultivar, and there were interacting effects between cultivar and photoperiod (**Table 4**). Clearly, the environmental influences on leaf P_net_ and g_s_ in potato can complex, and in certain instances change with age and cultivar.

### Photosynthetic Photon Flux Effects on Photosynthesis—P_net_

If effects of PPF, [CO_2_], and photoperiod are averaged for all ages and all three cultivars, some more clear comparisons might be drawn. Leaf P_net_ for all cvs. showed a 49% increase under 12-h and at 27% increase under 24-h photoperiod when PPF increased from 400 to 800 µmol m^−2^ s^−1^ at 350 ppm [CO_2_], and 40% increase at 12-h and at 12% increase at 24-h at 1,000 ppm [CO_2_] (**Figures 6** and **7**). This suggests that the benefits from increased PPF were reduced at elevated [CO_2_], and that doubling of the PPF from 400 to 800 µmol m^−2^ s^−1^ did not double the P_net_, which is not unexpected as the light intensities approach saturation levels. Dwelle et al. (1981; 1982) reported maximum photosynthetic rates for cv. Russet Burbank leaves occurring from 900 to 1,300 µmol m^−2^ s^−1^, Midmore and Prange (1992) reported maximum rates for cvs. DTO-33 and Guarhuash Huayro near 1,100 µmol m^−2^ s^−1^, and Paradiso et al. (2018) reported maximum rates for cvs. Avanti and Colomba near 1,500 µmol m^−2^ s^−1^. These are higher than 800 µmol m^−2^ s^−1^ used in our study, suggesting that photosynthesis for the cvs. in our study could have been increased even further with higher PPF. Some of the very first single leaf photosynthetic rates reported for potato, showed that P_net_ rates saturated near 585 µmol m^−2^ s^−1^ PPF at 1950 ambient [CO_2_] concentration of about 300 ppm, but that elevating the [CO_2_] to 600 ppm increased photosynthetic saturation to about 820 µmol m^−2^ s^−1^, and elevating [CO_2_] further to about 1,500 ppm increased photosynthetic saturation to about 1,015 µmol m^−2^ s^−1^ (Chapman and Loomis, 1953). Ku et al. (1977) reported that an irradiance of 850 µmol m^−2^ s^−1^ (~1/2 full sunlight) was saturating for potato leaves at ambient [CO_2_], but doubling the [CO_2_] could increase photosynthetic rates further.

### CO_2_ Effects on Photosynthesis—P_net_

Leaf P_net_ rates for all cultivars were increased with elevated [CO_2_] by 36% and 27% for 400 and 800 µmol m^−2^ s^−1^ PPF with a 12-h photoperiod, but decreased 11% and 20% with elevated [CO_2_] at 400 and 800 µmol m^−2^ s^−1^ PPF at 24-h lighting (**Figures 6** and **7**). The benefits of [CO_2_] enrichment on photosynthetic rates for the 12-h grown plants is consistent with numerous reports from other photosynthetic gas exchange studies (Chapman and Loomis, 1952; Sicher and Bunce, 2001; Bunce, 2003; Leakey et al., 2012; Fleisher et al., 2014; Kaminiski et al., 2014), but negative effect of [CO_2_] enrichment under 24-h lighting was somewhat unexpected. Prior leaf gas exchange measurements with potatoes grown under continuous light showed some benefit with [CO_2_] enrichment for cv. Norland early in growth, but there was little benefit after about 40 days age (Wheeler and Tibbitts, 1989). Single leaf P_net_ measurements for cv. Russet Burbank from that same study showed no benefit or even decreased rates throughout growth (Wheeler and Tibbitts, 1989), which is consistent with what we report here.

### Continuous (24-h) Light Effects

The results from this study suggest that continuous light was stressful for the potato leaves and plants over time, and that it reduced the beneficial effect of increasing the PPF or elevating the [CO_2_] on photosynthesis and biomass gain (Wheeler et al., 1991). Our intent in testing longer photoperiods, including continuous lighting, was to determine upper limits for potato tuber yield per unit area per unit time for space life support systems (Wheeler et al., 1986). For many studies, use of continuous light did indeed increase yields by providing a great daily light integral or DLI to the plants (Wheeler and Tibbitts, 1987; Wheeler et al., 1991; Wheeler and Tibbitts, 1997), but only with cultivars that were physiological tolerant to the continuous light. Depending on the cultivar, overall radiation use efficiency (gram dry mass/mol PAR) for tuber yields always tended to be lower with continuous light as well as other longer photoperiods, such as 16-h light/8-h dark or 20-h light/4-h dark (Wheeler et al., 1986; Demagante and Vander Zaag, 1988; Wheeler et al., 2008). This could be related to end-product inhibition, such as carbohydrate accumulation in the leaves under continuous light, especially with elevated [CO_2_] (Ehret and Jolliffe, 1985; Peet et al., 1986; Sicher and Bunce, 2001), or the fact that most potatoes tuberize better under short photoperiods (Batutis and Ewing, 1982; Wheeler and Tibbitts, 1986; Demagante and Vander Zaag, 1988). Any suppression of tuber initiation under continuous light may have limited potential carbohydrate sinks (i.e., tubers) from developing, and is consistent with the lower harvest index values reported from the same plants used in this study (Wheeler et al., 1991). In studies where potatoes where moved between environments with a 12-h photoperiod and continuous light, plants produced the greatest yields when they were started under short days, which presumably initiated a large number of tubers early in growth, followed by moving them to a continuous light environment, which provided more total light (Wheeler and Tibbitts, 1997). Unfortunately, no leaf photosynthetic measurements were taken for those studies.

### Stomatal Conductance

Elevated [CO_2_] reduced stomatal conductance, g_s_, for all the cultivars grown under the 12-h photoperiod for both PPFs in our study (**Figure 8**), which is consistent with numerous reports in the literature (Morison, 1987; Fleisher et al., 2012; Kaminski et al., 2014; Leakey et al., 2019). A comparison of g_s_ between potato plants grown at 8 h light/16 h dark, and 16 h light/8 h dark showed little difference (Ezekiel and Bhargava, 1992), but each of these environments provided a defined light/dark cycle for the leaf circadian entrainment (Holmes and Klein, 1986), unlike the continuous light grown plants in this study, which consistently showed lower g_s_ in comparison to 12-h light grown plants. Under the 24-h light treatment, elevated [CO_2_] actually increased conductance under 400 µmol m^−2^ s^−1^ PPF and decreased it only slightly under 800 µmol m^−2^ s^−1^, which was unexpected based on the volume of literature looking at [CO_2_] effects on stomata (Morison, 1987; Leakey et al., 2019).

If the effects of PPF on g_s_ are compared for all the cultivars over all dates, increasing the PPF from 400 to 800 µmol m^−2^ s^−1^ increased g_s_ by 22% at 350 ppm [CO_2_] and by 8% at 1,000 ppm [CO_2_] for plants grown under a 12-photoperiod (**Figure 8**). Under 24-h continuous light, increasing the PPF from 400 to 800 µmol m^−2^ s^−1^ increased g_s_ by 25% at 350 ppm [CO_2_] and by 23% at 1,000 ppm [CO_2_] (**Figure 9**). Increases in g_s_ with PPF are consistent with previous reports in the literature, including potato leaves (Vos and Oyarzun, 1987), and it is interesting to note that this occurred regardless of the photoperiod, unlike the [CO_2_] effects on g_s_ discussed above.

### Comparison of Leaf Photosynthesis and Yield

Total plant biomass data reported for this same study showed significant effects for [CO_2_], PPF, and cultivar, and significant interactions of [CO_2_] X cultivar and PPF X cultivar for plants grown under 12-h lighting (Wheeler et al., 1991). For plants grown under 24-h lighting, total biomass was affected by PPF and cultivar, with significant interactions between [CO_2_] X PPF and PPF X cultivar. Unfortunately, plant biomass yields for each photoperiod were analyzed separately and so the statistical effects of photoperiod were not determined. Leaf P_net_ rates for these same plants showed significant effects for [CO_2_], PPF, and photoperiod at 28 days after planting, and for PPF and photoperiod at 70 days after planting. But unlike total biomass and tuber yields, cultivar had no significant effect on leaf P_net_ rates (**Tables 1** and **2**).

When averaged for all cultivars, tuber yields increased in response to elevated [CO_2_] by 39% under the 12-h photoperiod and 400 µmol m^−2^ s^−1^ PPF (as compared to leaf P_net_ rates that were increased by 36%); tuber yields increased by 27% by elevated [CO_2_] at 800 µmol m^−2^ s^−1^ and a 12-h photoperiod (as compared to 27% increase for leaf P_net_); tuber yields increased 9% in response to elevated [CO_2_] under 24-h lighting at 400 µmol m^−2^ s^−1^ (as compared to a decrease in P_net_ by 11%); and tuber yields decreased 9% in response to elevated [CO_2_] under continuous light and 800 µmol m^−2^ s^−1^ PPF (as compared to a decrease in P_net_ 20%) (Wheeler et al., 1991). Similar trends occurred between total plant dry mass and upper canopy P_net_ rates (Wheeler et al, 1991). The greater deviation between upper canopy P_net_ rates and tuber biomass under continuous light could have been related to increased side lighting to the plants (Went, 1957), which were taller than the 12-h photoperiod plants. Likewise, the significant effect of cultivar on tuber and total biomass yields (Wheeler et al., 1991) but not on leaf photosynthetic rates (**Tables 1** and **2**) may have been related to shoot growth habits of the different cultivars (Norland plants tended to be shorter than Russet Burbank and Denali), which likely resulted in different amounts of side lighting. Thus our expectation that leaf photosynthetic rates are a good predictor of final yields for potatoes held for some, but not all combinations of light, CO_2_ and cultivar. The trends in leaf P_net_ for 12-h photoperiod plants seemed to match biomass and tuber yields better than for 24-h photoperiods. A better experimental approach would be to grow the plants in solid stands and only harvest plants surrounded by guard rows, or for limited space in controlled environments, use some form of side screening or shading to minimize complications from side lighting (Went, 1957).

### Implications for Space Life Support

The comparison of leaf P_net_ rates, and in particular, plant biomass yields for potato show that there is little benefit to increasing the PPF and [CO_2_] if continuous lighting is used, although increasing just PPF or [CO_2_] could provide some benefit. Stated differently, there is little benefit to using continuous light if you can [CO_2_] enrich and/or provide a higher instantaneous PPF. For all cases, radiation use efficiencies decreased under continuous light. Whether this is true for other long photoperiods (e.g., 16-h or 20-h) would require further study. Studies comparing tuber yields of cvs. Norland, Norchip, Superior, and Kennebec showed increased tuber yields with Norland at 16 and 20-h photoperiods at 400 µmol m^−2^ s^−1^ (Wheeler et al., 1986), but decreased yields for cv. Kennebec. This suggests that late season cultivars like Kennebec may be more obligate for short days to promote tuberization, while early season cultivars like Norland may be more day neutral with regard to tuberization (Wheeler et al., 1986). In addition, some cultivars like Kennebec, Superior and Norchip were physiologically intolerant to continuous light, a phenomenon that has reported with tomato and several other species (Hillman, 1956; Wheeler and Tibbitts, 1986).

When compared to terrestrial systems, space habitats and life support systems will all be constrained by mass, energy, and volume (Drysdale et al., 1999). To achieve a target crop yield and oxygen production could require higher light levels when growing area and volume are limited (Wheeler et al., 2008). Assuming that the crop yield is a response largely to the daily light integral, then this can be manipulated by increasing the instantaneous PPF, extending the photoperiod, or both. Assuming this would be done with electric lighting systems like LEDs, the total light produced would be constrained by the available electric power (Drysdale et al., 1999) (note, there are situations and approaches where solar lighting could be used in space; Nakamura et al., 2009). There can also be complications depending on the maximum output of the electric lighting fixtures and/or power allocations that might change throughout the day. Short day crops like potato can make things a bit more complex to manage if the longer photoperiods tend to suppress tuber development (likewise for short-day flowering of crops like soybean and rice); use of longer photoperiods can increase total biomass but harvest index (edible biomass/total biomass) might decrease. Moreover, if the crops are physiologically intolerant to very long photoperiods, as with some potato cultivars (Wheeler and Tibbitts, 1986), long photoperiods may not be an option. But we have seen a range of responses in potato cultivars with regard to photoperiod and tuberization (Wheeler and Tibbitts, 1986), and even tolerance to continuous light, so many of these challenges might be resolved by selecting appropriate genotypes or breeding/engineering for tolerance to longer photoperiods.

When we began these studies, we assumed the enriching the [CO_2_] to a level of 1,000 ppm would be beneficial for a C_3_ crop like potato because it would increase photosynthesis, growth, and water use efficiency, regardless of the lighting (Hand et al., 1993; Leakey et al., 2012; Leakey et al., 2019). Surprisingly, this was not the case under continuous light (Wheeler et al., 1991). [CO_2_] in closed atmospheres of spacecraft or habitats with humans is typically elevated well above current Earth ambient, where for example, the ISS [CO_2_] levels have ranged from 3,000 and 7,000 ppm (Law et al., 2014), so [CO_2_] enrichment should not be difficult to implement in space. Based on our findings with potato, this suggests that shorter photoperiods would be the most efficient approach to take advantage of the elevated [CO_2_] environment, and if energy for lighting is limited, the greatest benefits from [CO_2_] enrichment would occur at low to moderate PPF (e.g., 400 µmol m^−2^ s^−1^). These recommendations are focused on trying to optimize the environment to improve the performance of the crop. Alternatively, the crops, including potato, might be altered to accommodate the environment. This can be done through selection from existing genotypes, conventional plant breeding, or the use of genetic engineering approaches to develop better “space crops.”

## Data Availability Statement

The datasets generated for this study are available on request to the corresponding author.

## Ethics Statement

Written informed consent was obtained from the individual(s) for the publication of any potentially identifiable images or data included in this article.

## Author Contributions

TT was the senior investigator for this research and the NASA grantee. RW was postdoctoral fellow working with TT to study potatoes as a candidate crop for space life support systems. AF was a laboratory technician working for TT and assisted in making leaf photosynthetic measurements and data analysis.

## Funding

Research funded by a grant from NASA, US National Aeronautics and Space Administration.

## Conflict of Interest

The authors declare that the research was conducted in the absence of any commercial or financial relationships that could be construed as a potential conflict of interest.
